# What do you mean, you don’t know what is in the culture media?

**DOI:** 10.1016/j.xfre.2023.05.002

**Published:** 2023-05-12

**Authors:** Richard J. Paulson

**Affiliations:** Division of Reproductive Endocrinology & Infertility, Department of Obstetrics & Gynecology, Keck School of Medicine, University of Southern California, Los Angeles, California

A medical student visited our fertility center recently. She had lots of questions, and 1 of them addressed the various types of culture media that were being used for human embryos. I explained that a culture medium is simply a nutritive solution of electrolytes, glucose, and amino acids with a specific osmolality and pH and that when in vitro fertilization (IVF) started, the preparation and testing of culture media was a major part of the work of IVF laboratories. However, since we stopped making our own media more than 20 years ago, we no longer know the exact content of the various components. She looked incredulous, “you mean that you don’t know what is in the culture media?”

There is nothing like a naïve question to get one thinking. Indeed, how is it possible that physicians treating patients would not know the composition of a particular treatment that is being administered? We might consider preimplantation embryos to be our patients, and they are literally submerged in culture media for up to 7 days at a time. We certainly do consider the humans to whom we transfer the embryos to be our patients, and they are being injected with a small volume of culture medium during the embryo transfer process. Should we obtain informed consent from patients to be injected with some volume of an embryo transfer solution, the contents of which are not exactly known? Would we consider infusing a patient with an intravenous solution without knowing how much sodium or glucose it contained? If not, why are we tolerating the use of transfer media without the same information?

This is hardly the first time that the subject of culture media composition has been considered. Sunde et al. (1) eloquently called for action in 2016 after reviewing the literature regarding the influence of culture media on subsequent embryonic and fetal development. They stated, “published data indicate that there is a strong case for demanding full transparency concerning the compositions of and the scientific rationale behind the composition of embryo culture media.” That was 7 years ago, and we still do not have that information.

It is not impossible to find out what is in culture media. With today’s assay methods, it is conceptually simple to analyze solutions for their content of glucose, electrolytes, and amino acids. This analysis has been accomplished and published a number of times (2, 3, 4), and it is interesting to note how widely different culture media vary in their components. Morbeck et al. (2) found >100-fold differences in the concentration of various amino acids among different culture media, whereas the glucose content varied from 0 to 3.1 mM. A subsequent study by Morbeck et al. (3) evaluated the composition of 4 different single-step media. As in the earlier study, the composition of the media varied substantially. The glucose content varied from 0.18 to 0.97 mM, and there were large differences in the concentrations of electrolytes and amino acids. More recently, Tarahomi et al. (4) evaluated 15 different culture media. In addition to measuring the concentration of 37 components, they analyzed the stability of the media over time under conditions consistent with embryo culture. After their thorough analysis, they concluded that “companies are urged to fully disclose the components of their culture media, and provide clinical evidence supporting the composition of the media or future changes thereof, to facilitate advancement in this field of research” (4).

It seems clear that reasonable investigators agree that we should not be kept in the dark about the recipes that are being used to produce IVF culture media. Barnhart (5) pointed out that Lind’s evaluation of the choice of treatment of scurvy (in 1747) was evaluated with greater rigor than the choice of which culture medium to use in the 21st century. As clinicians, we should really take greater umbrage at having to inject into the uterine cavities of our patients a liquid of unknown composition. Yet, after repeated calls to action, nothing is changing. Producers of culture media continue to sell vials of varying composition without any disclosure of their content.

It is time for a new call to action. In the United Kingdom, the Human Fertilisation and Embryology Authority surely has the ability to mandate that all components of culture media be made public. In the United States, we rely on our professional organization, the American Society for Reproductive Medicine, and its affiliate, the Society for Assisted Reproductive Technology, to set standards and control the practice of IVF. It is time to set minimum standards for culture media based on all available data that we have. Inherent to those standards must be full disclosure of the content of all commercially available media. We can use the standard of 37 components measured by Tarahomi et al. (4) or perhaps just the 32 measured by Morbeck et al. (2). Either way, we must insist that continuing the use of culture media whose composition is not known is outside of the standard of care.
